# Association and Mutation Analyses of 16p11.2 Autism Candidate Genes

**DOI:** 10.1371/journal.pone.0004582

**Published:** 2009-02-26

**Authors:** Ravinesh A. Kumar, Christian R. Marshall, Judith A. Badner, Timothy D. Babatz, Zohar Mukamel, Kimberly A. Aldinger, Jyotsna Sudi, Camille W. Brune, Gerald Goh, Samer KaraMohamed, James S. Sutcliffe, Edwin H. Cook, Daniel H. Geschwind, William B. Dobyns, Stephen W. Scherer, Susan L. Christian

**Affiliations:** 1 Department of Human Genetics, The University of Chicago, Chicago, Illinois, United States of America; 2 Department of Neurology, The University of Chicago, Chicago, Illinois, United States of America; 3 Department of Pediatrics, The University of Chicago, Chicago, Illinois, United States of America; 4 Department of Psychiatry, The University of Chicago, Chicago, Illinois, United States of America; 5 Department of Psychiatry, Institute for Juvenile Research, University of Illinois at Chicago, Chicago, Illinois, United States of America; 6 Department of Molecular and Medical Genetics, The Centre for Applied Genomics and Program in Genetics and Genomic Biology, The Hospital for Sick Children, University of Toronto, Toronto, Ontario, Canada; 7 Department of Molecular Physiology and Biophysics, Vanderbilt University, Nashville, Tennessee, United States of America; 8 Program in Neurogenetics, Department of Neurology and Center for Autism Research and Treatment, The Semel Institute, David Geffen School of Medicine, University of California Los Angeles, Los Angeles, California, United States of America; 9 Committee on Neurobiology, The University of Chicago, Chicago, Illinois, United States of America; University of Wuerzburg, Germany

## Abstract

**Background:**

Autism is a complex childhood neurodevelopmental disorder with a strong genetic basis. Microdeletion or duplication of a ∼500–700-kb genomic rearrangement on 16p11.2 that contains 24 genes represents the second most frequent chromosomal disorder associated with autism. The role of common and rare 16p11.2 sequence variants in autism etiology is unknown.

**Methodology/Principal Findings:**

To identify common 16p11.2 variants with a potential role in autism, we performed association studies using existing data generated from three microarray platforms: Affymetrix 5.0 (777 families), Illumina 550 K (943 families), and Affymetrix 500 K (60 families). No common variants were identified that were significantly associated with autism. To look for rare variants, we performed resequencing of coding and promoter regions for eight candidate genes selected based on their known expression patterns and functions. In total, we identified 26 novel variants in autism: 13 exonic (nine non-synonymous, three synonymous, and one untranslated region) and 13 promoter variants. We found a significant association between autism and a coding variant in the seizure-related gene *SEZ6L2* (12/1106 autism vs. 3/1161 controls; p = 0.018). *Sez6l2* expression in mouse embryos was restricted to the spinal cord and brain. *SEZ6L2* expression in human fetal brain was highest in post-mitotic cortical layers, hippocampus, amygdala, and thalamus. Association analysis of *SEZ6L2* in an independent sample set failed to replicate our initial findings.

**Conclusions/Significance:**

We have identified sequence variation in at least one candidate gene in 16p11.2 that may represent a novel genetic risk factor for autism. However, further studies are required to substantiate these preliminary findings.

## Introduction

Autism (MIM 209850) is a phenotypically and etiologically heterogeneous disorder of childhood characterized by impairments in social interaction, deficits in verbal and non-verbal communication, and restricted interests and repetitive behaviors. Co-morbid features include mental retardation (occurrence ∼30–60%) [1], anxiety and mood disorders [2], and seizures (∼20%) [3]. Pathological and imaging studies indicate that structural brain abnormalities and aberrant synaptic connectivity may underlie the autism phenotype in some individuals [4], [5]. Autism comprises the severe end of a group of autism spectrum disorders (ASD), which also include Asperger syndrome, pervasive developmental disorder not otherwise specified (PDD-NOS), and rare syndromic forms including Fragile X and Rett syndromes [6]. Prevalence rates for autism and ASD are estimated at 0.2% and 0.6%, respectively, and males are more likely than females to have a diagnosis of ASD (male∶female ratio≈4∶1) [7], [8]. Twin and family-based studies indicate a strong genetic basis for autism [9], [10].

The frequency of microscopically visible structural chromosomal imbalances in autism is high and estimated at ∼7% [11], [12]. The most frequent abnormality is maternal duplication 15q11-13, which account for ∼1–3% of autism [13]. Other commonly observed cytogenetic abnormalities include deletions of 2q37, 22q11.2 and 22q13.3 [11], [14]. Recently, submicroscopic copy number variants (CNVs) not otherwise detectable using traditional cytogenetic techniques have been identified using whole-genome microarray-based approaches such as array comparative genomic hybridization (aCGH) and high-density SNP genotyping platforms [15]–[19]. Among these newly identified CNVs are microdeletions of 16p11.2, which have been observed in ∼0.5% of autism patients [18], [20], [21] making this the second most common chromosomal abnormality in autism. The reciprocal 16p11.2 microduplication has also been observed in ∼0.5% of autism patients, although the association is less convincing given a higher frequency in control cohorts [18], [20], [21]. Interestingly, the duplication has recently been found in ∼2.4% of patients with childhood-onset schizophrenia [22] as well as in ∼0.07% patients with bipolar disorder [21].

The 16p11.2 microdeletion/duplication spans ∼500 kb and is flanked by ∼147-kb low copy repeats (LCRs) that are >99% identical [20]. The intervening single copy sequence contains ∼24 genes and the flanking 147-kb LCRs contain at least three genes. Genomic losses (and possibly gains) at 16p11.2 could directly contribute to the autism phenotype by affecting dosage-sensitive genes in this region, disrupting genes at the breakpoints or unmasking recessive mutations on the other allele. In patients without these imbalances, functional sequence variants in one or more genes in 16p11.2 may represent risk factors for autism.

Two hypotheses have been proposed for the contribution of functional variants to common and complex human diseases such as autism [23]. The common disease common variant (CDCV) hypothesis postulates that common variants with small to modest effects (allele frequencies >1%) may underlie susceptibility to common disorders. Alternatively, the common disease rare variant hypothesis (CDRV) suggests that susceptibility to common disorders may be due to low frequency (∼0.01% to ∼1%) variants with moderate to high penetrance located in one or more genes [23]. The role of common and rare 16p11.2 sequence variants in the complex etiology of autism has not been examined. We therefore undertook a study of the 16p11.2 region to investigate the role of genetic variation in eight candidate genes we hypothesize may represent risk loci for autism spectrum disorders.

## Results

### Common genetic variation in 16p11.2

To assess whether common genetic variation at 16p11.2 might be associated with autism, we performed a family-based association analysis on two autism data sets that have recently been generated using two whole-genome high-density single nucleotide polymorphism (SNP) genotyping platforms. These microarray studies were performed on 777 families from the Autism Genetics Resource Exchange (AGRE) using the Affymetrix 5.0 array [21] and on 943 AGRE families using the Illumina Hap550 microarray (www.agre.org/) (Bucan and Hakonarson, unpublished). In addition, we analyzed data on 60 families generated from the Affymetrix 500 K platform [18].

We performed two types of tests: a transmission disequilibrium test (TDT) [24] with both parents genotyped and the DFAM test [25] using all families. The TDT identified one nominally significant association (p = 0.049) with intragenic marker rs7193756 (chr16:29,657,155-29,657,655) from the Affymetrix 5.0 data; however, the DFAM was not significant (p = 0.20) (Table S1). The association with rs7193756 and autism did not remain significant after correcting for multiple comparisons (region-wide p = 0.3576). The closest annotated gene to this marker is the transmembrane protein C16orf54 located ∼4-kb downstream of rs7193756. We used HapMap data (http://www.hapmap.org/) to generate linkage disequilibrium (LD) structure around rs719376, which indicated that it resides in an ∼56-kb block defined by markers rs9922666 and rs7205278 (chr16:29,605,876- 29,662,434) that contains the *QPRT* gene (data not shown).

### Rare genetic variation in candidate 16p11.2 genes

We next addressed whether rare variants in 16p11.2 genes represent risk factors for autism. We performed a literature review of the 24 genes at 16p11.2 and selected the following eight candidate genes for mutation analysis based on biological function, genetic mouse models, and expression data: *ALDOA* (NM_000034.2), *DOC2A* (NM_003586.2), *HIRIP3* (NM_003609.2), *MAPK3* (NM_002746.2), *MAZ* (NM_002383.2), *PPP4C* (NM_002720.1), *SEZ6L2* (NM_201575.2), and *TAOK2* (NM_004783.2) (Table S2). For each of the eight candidate genes, we sequenced the coding regions and their associated splice sites, 5′ and 3′ untranslated regions (UTRs), and proximal promoter region (∼1500 bp upstream of the transcription start site) in an initial minimum sample of ∼100 unrelated autism subjects. In parallel, we sequenced all eight candidate genes in ∼100 control subjects to assess the natural genetic variation at these loci and to identify autism-specific variants. We also sequenced these genes in five previously reported patients with 16p11.2 microdeletions [20] to test the hypothesis that microdeletions might unmask recessive alleles on the non-deleted chromosome. In total, we identified 26 novel, autism-specific rare variants, including 13 exonic and 13 promoter variants (Table 1). A complete description of these variants, including demographic data on patients, and conservation, transmission and segregation of each variant, is presented in Table S3. We also identified 44 control-specific variants in the eight genes examined (Table S4).

**Table 1:** Summary of exonic and promoter variants identified in autism.

Gene	Location^a^	Amino Acid^b^	Nucleotide^c^	Genotype^d^	Chromosome^f^	Protein Prediction^g^	Autism Frequency^k^	Control Frequency^k^
ALDOA	5′ UTR	na	123244G>A	Het	chr16:29974016	na	1/86	0/92
DOC2A	Pro2	na	77883G>A	Hom	chr16:29930371	na	1/85	0/89
DOC2A	Exon 6	M225I	82362G>A	Het	chr16:29925892	Possibly damaging	1/176	0/258
DOC2A	Exon 7	R266Q	82566G>A	Het	chr16:29925688	Benign	1/176	0/258
HIRIP3	Pro2	na	92636G>A	Het	chr16:29915618	na	1/91	0/93
HIRIP3	Pro5	na	93598T>C	Het	chr16:29914656	na	1/85	0/93
HIRIP3	Exon 5	R419C	61843C>T	Het	chr16:29912615	Benign	1/179	0/183
MAZ	Pro2	na	114496T>G	Het	chr16:29724343	na	1/92	0/90
PPP4C	Pro2	na	14556T>C	Het	chr16:29993698	na	1/92	0/90
PPP4C	Pro2	na	14708T>C	Het	chr16:29993546	na	1/92	0/90
MAPK3	Exon 2	V63M	133587G>A	Het	chr16:30040810	Benign	1/91	0/184
SEZ6L2	Pro3	na	62867G>A	Het	chr16:29818551	na	1/91	0/91
			63078G>A	Het				
SEZ6L2	Pro4	na	63078G>A	Hom	chr16:29818340	na	2/86	0/86
SEZ6L2	Exon 2	E38E	64646G>A	Hemi^e^	chr16:29816772	na	1/708	0/556
SEZ6L2	Exon 3	T77T	65494G>A	Het	chr16:29815924	na	1/527	0/278
SEZ6L2	Exon 7	S396L	74926C>T	Het	chr16:29806492	Benign	1/1099	0/1152
SEZ6L2	Exon 11	P588L	85179C>T	Het	chr16:29796239	Benign	1/527	0/278
SEZ6L2	Exon 13	P724L	88933C>T	Het	chr16:29792485	Benign	1/527	0/554
SEZ6L2	Exon 13	L734Q	88963T>A	Het	chr16:29792455	Benign	1/527	0/554
SEZ6L2	Exon 16	I887I	90367C>T	Het	chr16:29791051	na	1/527	0/278
TAOK2	Pro2	na	117019G>A	Het	chr16:29891235	na	1/91	0/93
TAOK2	Pro2	na	117050G>A	Het	chr16:29891204	na	2/183	0/457
TAOK2	Pro5	na	115685C>G	Het	chr16:29892569	na	1/91	0/93
TAOK2	Pro5	na	115775G>A	Het	chr16:29892479	na	1/91	0/93
TAOK2	Exon 14	A522T	104078G>A	Het	chr16:29904176	Benign	1/87	0/83

a UTR, untranslated region; Pro, promoter (specific nucleotide position is specified in Table).

b Positions based on the following accession numbers: DOC2A (Q14183); HIRIP3 (Q9BW71); MAPK3 (P27361); SEZ6L2 (Q6UXD5); TAOK2 (Q9UL54).

c Positions based on the following accession numbers: ALDO, DOC2A, HIRIP3, and PPP4C (AC093512.2); MAZ (AC009133); MAPK3 (AC012645.7); SEZ6L2 (AC120114.2); TAOK2 (AC093512.2).

d Het, heterozygous; Hom, homozygous; Hemi, hemizygous.

e This patient harbors a 16p11.2 microdeletion.

f Based on UCSC Genome Browser (http://genome.ucsc.edu/), Human March 2006 Assembly.

g Based on PolyPhen (http://genetics.bwh.harvard.edu/pph/).

k Number of individuals.

### Genetic variation in SEZ6L2 is associated with autism

Our most interesting preliminary finding was a recurrent autism-specific R386H amino acid substitution in exon 7 of the seizure-related gene *SEZ6L2* that we identified in our initial mutation screen (4/93 autism and 0/93 controls). This association was not significant with these low numbers but suggested a trend (Fisher's exact two tailed p = 0.12). We determined inheritance and segregation of R386H and demonstrated perfect segregation of this variant with the autism phenotype in all four families. This variant was not predicted to affect protein function using PolyPhen; however, R386H results in a strongly basic (arginine) to weakly basic (histidine) substitution in the CUB domain that is found almost exclusively in extracellular and plasma membrane-associated proteins, many of which are developmentally regulated [26], [27]. The R386 residue was conserved in 16 representative mammalian species and was present as H386 in five representative fish species (http://genome.ucsc.edu/; hg18). The overall initial pattern of association and specificity to autism as well as the potential role of *SEZ6L2* in seizures (which are present in ∼20% of autism cases) warranted further analyses of this gene.

We undertook a case-control association analysis of R386H by sequencing exon 7 in an additional 1013 autism patients and 1068 controls. The majority of these subjects were of European descent (Table S5). Among all individuals studied, we found a statistically significant association between R386H and autism (12/1106 autism versus 3/1161 controls; Fisher's exact two tailed p = 0.018). All 15 subjects harboring R386H were of European descent. In all cases, the variant was inherited with no bias between maternal versus paternal transmission (Table S6).

We performed a phenotype analysis on patients with R386H. None of the probands or any of their affected siblings were reported to have seizures. No common phenotypic features were observed among subjects with R386H.

We performed a replication study of R386H in independent autism and control cohorts, which failed to replicate our initial findings (4/529 autism versus 9/570 control; Fishers exact two tailed p = 0.42). Unexpectedly, five of the nine controls carrying the R386H variant had ancestry in the Orkney Islands, suggesting a possible founder effect. When all families with ancestry from the Orkneys were excluded from the control cohort, the findings were still not replicated.

To look for additional rare variants in *SEZ6L2*, we sequenced the remaining 16 exons in an additional 434 autism patients and 185 controls and identified seven autism-specific coding variants (four non-synonymous and three synonymous) and two promoter variants (Table 1). Examination of the autism-specific variants in parents and affected and/or unaffected siblings demonstrated that all variants were inherited. We identified a synonymous substitution in the middle of exon 2 (E38E) in a patient (HI2997) previously reported to harbor a 16p11.2 microdeletion [20], [21]. We cannot exclude the possibility that this substitution might affect *SEZ6L2* regulation, as synonymous mutations can occasionally affect protein function by altering mRNA stability and protein synthesis [28].

### Mouse and human expression studies of SEZ6L2

**Figure 1:**
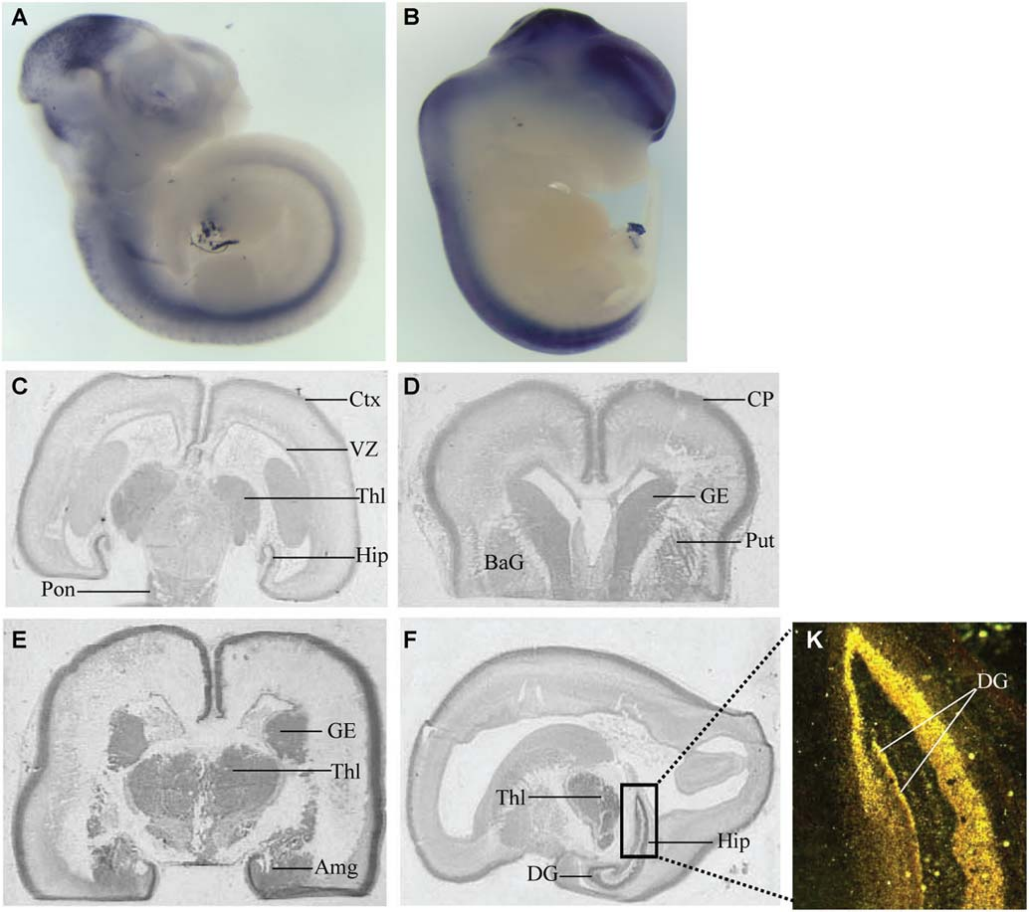
*SEZ6L2* is expressed in mouse and human central nervous systems. *In situ* analyses in whole mouse embryos demonstrate that *Sez6l2* mRNA is expressed in the developing brain and spinal cord at e10.5 (A) and e12.5 (B). Human *SEZ6L2* transcript distribution was assayed in 18- and 19-week-old human brains sectioned in either coronal orientation in subjects 1137 (C), 1110 (D, E) and in the sagittal orientation in subject 4889 (F). *SEZ6L2* is enriched in the cortical plate in the post mitotic neuron, in the ventricular zone, in the hippocampus, thalamus, ganglionic eminence, basal ganglia, and amygdala and at lower levels in the pons and the putamen. Emulsion picture of the dentate gyrus showing cellular specificity within the hippocampus (K). Sense controls tested on adjacent sections (not shown) gave no signal. Am, Amygdala; BG, Basal ganglia; CP, Cortical plate; DG, Dentate gyrus; GE, Ganglionic eminence; Hi, Hippocampus; Pu, Putamen; Th, Thalamus.

We performed *in situ* hybridization studies of *SEZ6L2* in mouse embryos and human fetal brains. In mouse embryonic day 10.5 (e10.5) and e12.5 embryos, *Sez6l2* expression was restricted to the brain and spinal cord (Figure 1A–B). We also reviewed the GENSAT mouse brain expression database (http://www.gensat.org). At e15.5, *Sez6l2* expression was highest in the olfactory bulb, cerebellum, and brainstem; at postnatal day 7, expression was widely distributed throughout the brain at low levels. Analyses of human fetal brains (gestational weeks 16–19) showed high *SEZ6L2* expression in post-mitotic cortical layers, hippocampus, basal ganglia, amygdala, thalamus and at lower levels in the pons and putamen (Figure 1C–K). The developmental expression pattern of *SEZ6L2* in mice and humans is consistent with the neurodevelopmental basis of autism spectrum disorder [29], thereby providing further support for a role of *SEZ6L2* in autism.

### Genetic variation in other 16p11.2 genes

We identified 16 autism-specific rare variants in seven additional 16p11.2 candidate genes analyzed (Table 1) and 33 control-specific variants (Table S4). Of the autism-specific variants, five were coding (all non-synonymous), one was located in the 5′ UTR, and ten were promoter variants. One of the coding variants, M225I, was identified in the synaptic vesicle gene *DOC2A* and was predicted to alter protein function. This paternally inherited variant was also present in an affected sibling but absent in an unaffected sibling. In addition, M225I was absent in 258 control subjects. The ethnicity of the patient harboring M225I was indicated as ‘White - Hispanic or Latino’ and most of our controls are of European descent with no specific information on Hispanic or Latino ancestry. Therefore, M225I might represent a Hispanic/Latino-specific variant.

For the promoter variants, we determined nucleotide conservation across several species, performed transcription factor binding site (TFBS) analyses, and determined transmission and segregation patterns in families (Table S3 and Table S7). One substitution of interest was a g.77883G>A change found in the *DOC2A* promoter region. This variant was predicted to alter binding sites for several transcription factors that have established roles in brain and behavioral development.

We formally assessed the mutation burden of rare variants in patients versus controls, but did not detect a statistically significant difference in the total number of autism-specific variants compared to control-specific variants (Fisher's exact two tailed p = 0.42). We stratified our analyses by gene as well as by coding and promoter regions, and did not detect any significant differences in variant frequencies between patients and controls.

## Discussion

We undertook a study of the 16p11.2 microdeletion/duplication region to investigate the role of common and rare genetic variation in 16p11.2 loci and risk for autism. Common and complex diseases such as autism can be due to genetic variation associated with a wide spectrum of allele frequencies [30]. We hypothesized that common and/or rare functional variants in one or more genes in 16p11.2 may confer susceptibility to autism. To elucidate the potential role of 16p11.2 common genetic variation in autism, we analyzed existing SNP genotyping data from the following platforms: Affymetrix 5.0, Illumina 550 K, and Affymetrix 500 K microarrays. Our analysis identified a single nominal association with rs7193756 that resides in a LD block that contains the transmembrane protein *C16orf54* and the quinolinate phosphoribosyl-transferase gene *QPRT*. Overall, our association analyses indicate that common variation at 16p11.2 is not a major risk factor in autism. However, we cannot rule out the possibility that common (functional) variants not represented on the three commercially available microarrays may be associated with autism.

We also hypothesized that one or more genes residing within the 16p11.2 region harbor rare variants that increase risk for autism. In other studies, systematic mutation screening of genes initially identified through chromosomal, CNV, and/or resequencing analysis has led to the discovery of rare autism-associated variants/mutations in several genes, including *NLGN3* and *NLGN4* at Xp22.3 [31], *NRXN1* at 2p16.3 [15], [32], *SHANK3* at 22q13 [33]–[35], and *CNTNAP2* at 7q35 [36]–[38]. In the present study, we identified an initial significant association between a novel *SEZ6L2* coding variant R386H and autism (p = 0.014). *SEZ6L2* is an intriguing candidate given the increased risk of clinical or subclinical epilepsy in autism (∼20% of patients) [3]. *SEZ6L2* is referred to as a seizure-related gene because a closely related ortholog, *Sez-6*, is upregulated in response to seizure-inducing reagents in mouse neurons [39]. The R386H substitution resides within a CUB domain that is found in functionally diverse developmental proteins such as Tolloid (involved in dorso-ventral patterning) and A5 (critical for targeting growing axons during nerve innervation) [27]. Our expression studies of mouse and human *SEZ6L2* in the developing embryo demonstrated high CNS-specific levels of brain expression, as would be expected for a neurodevelopmental disorder such as autism [40]. Mice deleted for *Sez6l2* do not show any obvious defects in development or behavior [41]. However, mice deleted for all three *SEZ* family members exhibit abnormal behavior that includes impaired motor coordination [41]. It is possible that R386H may be necessary but not sufficient to produce autism and related disorders in some patients. Although the data presented here are insufficient to implicate a clear role for R386H in autism, follow-up investigations such as additional replication studies and functional experiments are warranted to evaluate its importance in disease risk.

Our screen for rare variants in seven additional genes identified several nucleotide substitutions of potential interest. The M225I substitution, predicted to affect protein function, was identified in the brain-specific synaptic vesicle-associated protein DOC2A (Double C2-Like Domain-Containing Protein, Alpha) that is thought to serve as a calcium sensor in neurotransmitter release [42], [43]. The M225I substitution is located between the two C2 domains, which interact with Ca^2+^ and phospholipids. Mice deleted for *Doc2a* show alterations in synaptic transmission and long-term potentiation and exhibit learning and behavioral deficits that include an abnormal passive avoidance task [44]. We also identified a *DOC2A* promoter variant in another patient that is predicted to alter transcription factor binding sites for several brain-expressed genes.

In conclusion, we report an initial analysis of common and rare genetic variation in the 16p11.2 microdeletion/duplication region that is associated with ∼1% of autism cases. The novel rare variants identified in this study represent an initial catalog of low frequency, putative functional risk factors in autism. We do not report compelling evidence for a role of either common or rare genetic variants in autism etiology. Our findings might be interpreted to suggest that deletion and/or duplication of multiple genes in the 16p11.2 interval is a more significant genetic risk factor for predisposition to autism, rather than molecular risk contributed by any one gene at this locus. Given that our choice of eight candidate genes was somewhat biased towards biological function, it is also possible that other genes or genomic features in the 16p11.2 region might contribute to autism. Although mutations associated with autism have been identified by screening as few as several hundred patients [32], [33], one limitation of our study is the relatively small number of patients screened for rare variants. Additional studies in a larger number of patients for the genes examined here are warranted. In addition, the application of next-generation sequencing strategies to screen all genes and regulatory elements within the microdeletion/duplication may reveal more significant abnormalities.

## Materials and Methods

### Ethics Statement

All research involving humans and animals have been approved by the Institutional Review Boards of The University of Chicago, The University of Toronto, and The University of California, Los Angeles. All families provided written informed consent for the collection of samples and subsequent analysis.

### Autism and Control Subjects

Genomic DNA from autism and control subjects were obtained from various sources as described below. Ethnic breakdown for all autism and control groups are provided in Table S5. We obtained autism samples from several DNA repositories including the Autism Genetics Resource Exchange (AGRE) (n = 793) and the National Institutes of Mental Health (NIMH) (n = 313). For the AGRE sample set, the Autism Diagnostic Interview–Revised (ADI-R), Autism Diagnostic Observation Schedule (ADOS), Raven and Handedness, Peabody and Vineland assessments were performed. Medical histories and physical neurological exams were also collected. Additional phenotypic data on the AGRE sample set are available on the AGRE website (http://www.agre.org/). Genomic DNA from control subjects were obtained from the NIMH Genetics Initiative Control sample set (n = 1161); these subjects were screened for any Axis I mental health disorders and none had a diagnosis of autism.

Genomic DNA was also obtained from several Canadian institutions including The Hospital for Sick Children in Toronto and in child diagnostic centers in Hamilton, Ontario, and in St. John's, Newfoundland (n = 529). For the Canadian autism cohort, all subjects met ADI-R and ADOS criteria conclusively or on a clinical best estimate. Most index patients (∼75%) were screened for fragile×mutations and were karyotyped. Wherever possible, experiments were performed on blood-derived genomic DNA (80%); otherwise, DNA from cell lines was used. Control DNA was isolated from cell lines from the Ontario Population Genomics Platform (n = 570). Subjects living in Ontario, Canada were recruited by telephone from a list of randomly selected residential telephone numbers for Ontario and from population-based Tax Assessment Rolls of the Ontario Ministry of Finance. Health and Ancestry of these subjects is self reported in an extensive questionnaire.

### Association analyses

Association analyses were performed on existing data generated on the following three SNP genotyping platforms: 1) Affymetrix 5.0 data available on 777 AGRE families by the Autism Consortium [21]; 2) IlluminaHap550 data available on 943 AGRE families by the microarray facility at Children's Hospital of Philadelphia (www.agre.org/) (Bucan and Hakonarson, unpublished); and 3) Affymetrix 500 K platform data on 60 families [18]. PLINK v1.03 was used for the analysis [45]. Two different types of analyses were performed. First, we performed the transmission disequilibrium test (TDT) [24] with permutation for families with 2 genotyped parents and 1 or more affected offspring. The permutation procedure flips transmitted/untransmitted status constantly for all SNPs for a given family, thereby preserving the linkage disequilibrium and linkage information between markers and siblings. Second, we used DFAM for all individuals. DFAM within PLINK implements the sib-TDT [25] and also allows for unrelated individuals to be included (via a clustered-analysis using the Cochran-Mantel-Haesnzel) and can be used to combine discordant sibship data, parent-offspring trio data and unrelated case/control data in a single analysis. Region-wide significance for both tests was estimated using the mperm option in PLINK which uses permutation to correct for multiple testing of all the markers within the region while taking linkage disequilibrium into account.

### DNA amplification and sequencing

Genes (accession numbers) examined in this study include: *ALDOA* (NM_000034.2), *DOC2A* (NM_003586.2), *HIRIP3* (NM_003609.2), *MAPK3* (NM_002746.2), *MAZ* (NM_002383.2), *PPP4C* (NM_002720.1), *SEZ6L2* (NM_201575.2), and *TAOK2* (NM_004783.2). PCR-amplification primers were designed using Primer3 (http://frodo.wi.mit.edu/) with M13 Forward and Reverse Tails added to each primer to facilitate high-throughput DNA sequencing (Table S8). DNA was amplified in a reaction comprised of: 20 ng genomic DNA, 1× buffer I (1.5 mM MgCl2, Applied Biosystems, Foster City, CA), 1 mM dNTPs (Applied Biosystems), 0.4 µM primer (each of forward and reverse, IDT, Coralville, IA), and 0.25 units AmpliTaq Gold (Applied Biosystems) in a total volume of 10 µl. Thermocycling conditions were as follows: 94°C for 10 min; 35 cycles of 94°C for 30 sec, annealing temperature (53–60°C) for 30 sec, and 72°C for 30 sec; and final extension of 72°C for 10 min. Variations in reaction composition and cycling conditions were required for a small number of amplicons. PCR products were purified in a 10 ul reaction comprised of 6.6 units Exonuclease I and 0.66 units shrimp alkaline phosphatase that were incubated at 37°C for 30 min followed by 80°C for 15 minutes. Sequencing reactions were performed using Big Dye terminators on an ABI 3730XL 96-capillary automated 3730XL DNA sequencer (Applied Biosystems) at The University of Chicago DNA Sequencing and Genotyping Core Facility. Sequence data were imported as AB1 files into Mutation Surveyor v3.10 (SoftGenetics, State College, PA). Sequence contigs were assembled by aligning the AB1 files against GenBank reference sequence files that were obtained from the National Center for Biotechnology Information (NCBI) (http://www.ncbi.nlm.nih.gov/Genbank/). Reference sequences included the complete 5′ untranslated region (UTR), coding sequence and associated splice-sites, intronic sequence, and complete 3′ UTR. The imported GenBank files provide annotated features for each gene that include base count, intron/exon boundaries, amino acid sequence, and previously reported mutations and single nucleotide polymorphisms (SNPs) from the SNP database (dbSNP) (http://www.ncbi.nlm.nih.gov/projects/SNP/). To screen for putative mutations, the entire length of the sample trace was manually inspected for quality and variation from the reference trace. All detected variants were visually reviewed by two trained individuals and were confirmed using bi-directional sequencing.

### Human and mouse SEZ6L2 expression studies

Mouse *in situ* hybridization experiments were performed as previously described [46] on wildtype CD-1 whole embryos using DIG-labeled RNA probe for *Sez6l2* (IMAGE clone 6467632, Invitrogen). Human *in situ* hybridization experiments were performed on fresh frozen post-fixed tissues as previously described. [47]. The *SEZ6L2*-specific sequence (MHS1011-59266) was obtained from OpenBiosystem (Huntsville, AL), sequenced for verification, and checked for specificity with BLAST against the human genome. *In vitro* transcription was then performed to generate S^35^-labeled cRNA. Labeled cRNA was hybridized on 20 µm thick cryostat frozen tissue sections, sectioned into either coronal or sagittal plane and opposed to autoradiography films for two to five days. Slides were then coated with NTB2 autoradiography emulsion (Kodak, New Haven, CT), exposed for four weeks, and developed. Following staining with cresyl violet, emulsion dipped slides were cover-slipped and imaged using Nikon Eclipse E600 microscope with a Digital Capture System built around spot cooled CCD camera. Corresponding sense probes were used on sections adjacent to those used for antisense probes.

### Bioinformatic and statistical analyses

Gene selection was performed using the UCSC Genome browser (http://genome.ucsc.edu/) and literature review of articles published in PubMed (http://www.ncbi.nlm.nih.gov/sites/entrez). PolyPhen was used to predict whether amino acid substitutions affect protein function. The differences in frequency of any variant between cases and controls were assessed using the Fishers Exact test.

## Supporting Information

Table S1 Family-based association analyses of 16p11.2 markers in autism
(0.08 MB XLS)

Table S2 Candidate 16p11.2 genes selected for mutation analyses
(0.14 MB XLS)

Table S3 Complete summary of exonic and promoter variants identified in autism
(0.04 MB XLS)

Table S4 Control specific variants identified in eight candidate 16p11.2 genes
(0.04 MB XLS)

Table S5 Ancestries of autism and controls subjects used in mutation screen and association analyses
(0.03 MB DOC)

Table S6 Inheritance and segregation analysis in patients with R386H
(0.02 MB XLS)

Table S7 Promoter variants identified in autism patients
(0.05 MB XLS)

Table S8 PCR primers used to amplify candidate genes on 16p11.2
(0.11 MB XLS)
