# Supplementary material for: Association and Mutation Analyses of 16p11.2 Autism Candidate Genes
Source: PLoS One. 2009 Feb 26;4(2):e4582. doi: 10.1371/journal.pone.0004582 (PMC2644762; doi:10.1371/journal.pone.0004582)
Supplement: Table S5 — Ancestries of autism and controls subjects used in mutation screen and association analyses (0.03 MB DOC) [file pone.0004582.s005.doc]

**Table S5. Ancestries of autism and controls subjects used in mutation screen and association analyses**

| Group | Source^b^ | White | Hispanic or Latino | Black or  African American | Asian | Pacific Islander^c^ | More than one Race | Unknown |
| --- | --- | --- | --- | --- | --- | --- | --- | --- |
| Autism (n= 800) | AGRE | 546 (68.25%) | 99 (12.38%) | 23 (2.88%) | 16 (2.00%) | 3 (0.38%) | 52 (6.50%) | 11 (1.38%) |
| Autism (n= 313) | NIMH | 277 (88.5%) | 10 (3.19%) | 10 (3.19%) | 7 (2.24%) | - | - | 9 (2.9%) |
| Autism (n = 529)^a^ | Canada | 436 (82.5%) | - | 3 (0.5%) | 21 (4.0%) | - | 69 (13%)^d^ |  |
| Controls (n = 1162) | NIMH | 1060 (91.2%) | - | 102 (8.78%) | - | - | - | - |
| Controls (n = 570) | Canada | 570 (100%) | - | - | - | - | - | - |

^a^ Estimates based on Affymetrix 500K array

^b^ Mutation screening and initial association analyses of R386H were performed using the AGRE and NIMH samples whereas the replication study of R386H was performed using the Canadian samples

^c^ Includes one individual reported as Hispanic or Latino

^d^ Mostly European in origin
